# Dr. Julius Kratter on the Elimination of Strychnia through the Urine

**Published:** 1882-05

**Authors:** Julius Kratter

**Affiliations:** Docent of Hygiene, University of Gratz


### Original Translations.

---

ARTICLE XVIII.

### DR. JULIUS KRATTER ON THE ELIMINATION OF STRYCHNIA THROUGH THE URINE. Translated from the German by F. H. GENTSCH, M.D., Chicago.

Many of the most important alkaloids, whether taken by mouth or hypodermic injection, are very radidly absorbed and taken into the circulation, and without change again expelled by the urine. With the most of them the elimination follows very rapidly, and can often be demonstrated in the urine in a very short time after being taken into the system.

This is known to a certainty of the volatile alkaloids conium and nicotine, and a list of the fixed vegetable basis. Dragendorf demonstrated by experiments upon animals, the rapid elimination of the unaltered veratrim and aconite.

That atrofine is eliminated through the urine is well-known and has been demonstrated upon the human subject as early as 1810. In three cases of poison by stramonium seeds, Allen found atropia in the urine. Cohn and Körner record the same in a case of atropia poison after analysis by Harley. According to Menot and Schmidt, the elimination by the urine is soon completed, as in the first of the above cases, after three hours, and in the latter, after ten hours, no more atrofine was found in the urine. This property of atrofine was frequently demonstrated by Dragendorff, and the same was found true of hyosciamin. So also of the much used therapeutic agent morphia, is it is well-known, that it leaves the body after a short interval of time after its ingestion, through the urine. The older investigators, such as Lassagne, Olivier and Mooge, and finally Orfila and numerous later researches by Taylor, Bauchardat, Lefort, Kautzmann, Dragendorff, and others, confirmed the results of the older investigators. I, myself, found it in the urine eight or ten hours after death by suicide, whilst at the same time I found no trace of it in the stomach. Lewenstein made good use of these well-known facts for the detection of secret morphine eaters.

Not so unanimous, however, are the statements in regard to strychnia. That it is absorbed and is present in the blood was demonstrated as early as 1827 by Verniere. He took the blood of animals poisoned by the nux vomica bean, and poisoned others with it. The presence of strychnia in the blood and viscera was first demonstrated by Palmer in 1855. Taylor was unable to show the presence of strychnia in the corpse of Jno. Pearson's cook, the victim of Dr. Palmer. During the defense, the chemist, Herepath, testified that it was possible to detect 1-50,000 part of a grain of strychnia in the viscera. It was first clearly shown in the blood of the human subject by Oystow, later by M. Adam in the blood, muscles and urine, and by Auder seen in the liver. It seems thus to be practically demonstrated that we may accept the same law for the deportment of strychnia in the human organism in this regard as for morphine and the other alkaloids.

From the experiments of Cloötten and Dragendorff upon the lower animals, we learn:

1st. That strychnia, whether given by the stomach or subcutaneously, is rapidly absorbed.

2nd. That it finds its way into the blood unchanged, and is eliminated again unchanged. Unlike morphia, however, strychnia is not so rapidly eliminated, but slowly, and does not begin to pass off until several days after its ingestion, but is retained in the liver, so that in acute cases of poisoning by strychnia the urine is to be left out of consideration.

In all chronic cases of strychnia poison the poison is cast off from the body through the urine, but it must be remembered that the eliminating begins later and is carried on very slowly. This theory is propounded also in the various text-books on texicology. F. A. Falk, text-book on Practical Texicology Stuttgart, 1880, and Dragendorff on Poisons, St. Petersburg, 1876, may be recognized as understood and well authenticated, but we find in the literature records of experiments, which do not agree with it. M. Adam found in one case strychnia in the urine in nine minutes after the poison had been taken. A. O. Schulzen (Aschines, Auot and Phyiol, 1864,) and Rodgers found it in the urine in acute case of strychnia poisoning in the human subject. In one case, after seven hours chloroform inhalation, with recovery, Hamilton found it in the urine. Dragendorff, in company with Weyrich, found it in the urine after two days.

These contradictory statements in regard to the deportment of strychnia in the human organism, led me in a case of suicide by strychnia poison, in November, 1879, to give particular attention to the examination of the urine. Death followed just one hour and a half after the taking of the poison; 200 c.c. of urine of acid reaction was drawn, per catheter, from the bladder, in which I found, by analysis, in the laboratory of Prof. Schonenstein, unmistakable evidence of the presence of strychnia. In this case the poison was taken in considerable quantities in the solid form, as the undissolved strychnia was still found in the stomach on post mortem examination.

Here, then, we find a case of strychnia poison in the human subject, all the details and features of which are well-known, where, after the short period of one hour and thirty minutes the urine was found to contain the poison, a result at direct variance with the Masing-Dragendorff theory, which is based upon the results of experiments upon the lower animals alone.

DR. JULIUS KRATTER,
Docent of Hygiene, University of Gratz.

*Miner Medizinische Wochensctrift, Feb.* 25, 1882.

---

DEAFNESS IN CHILDREN.—Dr. Weill, of Stuttgart, found as a result of his examination of 4,500 school children, that thirty per cent. of them have imperfect hearing on one side, and that the defective hearing increases with age.—*Vienna Neue Freie Presse.*

